# Higher THC Concentration Medicinal Cannabis Products Efficacy and Safety Considerations: A Rapid Review

**DOI:** 10.1111/dar.70145

**Published:** 2026-04-01

**Authors:** Myfanwy Graham, Dereje Assefa, Ngo Cong‐Lem, Suzanne Nielsen

**Affiliations:** ^1^ Monash Addiction Research Centre Eastern Health Clinical School, Monash University Melbourne Australia; ^2^ Monash Sustainable Development Institute Evidence Review Service Monash University Melbourne Australia

**Keywords:** adverse drug event, delta‐9‐tetrahydrocannabinol, efficacy, medicinal cannabis, safety

## Abstract

**Issues:**

Higher delta‐9‐tetrahydrocannabinol (THC) concentration medicinal cannabis products are characterised by a higher THC and minimal cannabidiol (CBD) content. This rapid review aims to systematically summarise and evaluate the available evidence regarding the efficacy and safety of higher THC potency medicinal cannabis products relevant to the Australian market in adult populations.

**Approach:**

The rapid literature review protocol was prospectively registered with Open Science Framework: https://doi.org/10.17605/OSF.IO/HNFUT. The comprehensive search (1‐January‐2014–30‐July‐2024) included Medline, EMBASE, EMCARE, CINAHL, SCOPUS and the Cochrane Central Register of Controlled Trials databases. Eligible studies (randomised controlled trials and observational studies) were screened by two independent reviewers and then extracted.

**Key Findings:**

We identified 9969 records, resulting in 15 studies (six RCTs and nine observational), with THC concentrations of 16%–22% that met inclusion criteria. Studies examining efficacy for pain (*n* = 4), ulcerative colitis (*n* = 1) and chronic obstructive pulmonary disease (*n* = 1) reported mixed outcomes. Reported adverse events included psychiatric, nervous system and gastrointestinal effects. Validated cannabis use disorder (CUD) screening tools were notably absent from the included studies.

**Implications:**

Few studies have assessed products equivalent to the Australian Category 5 medicinal cannabis product definition, with most having THC concentrations lower than those available for prescription in Australia, including products reported to contain up to 88% THC. High‐quality RCTs and longitudinal studies that incorporate validated CUD screening tools are needed.

**Conclusion:**

No evidence was found on safety or efficacy of prescribed Category 5 medicinal cannabis products with THC concentrations above 22% w/w (220 mg/g).

## Key Point Summary

- There are a limited number of randomised controlled trials (RCT) for higher delta‐9‐tetrahydrocannabinol (THC) concentration products consistent with Category 5 products in the Australian regulatory setting.
- Products with up to 22% w/w THC 220 mg/g (range 16% to 22%) were identified in research, which is far lower than available products in Australia reported to contain up to 88% THC.
- Many of the available RCTs are single dose or short duration studies, which report statistically but not clinically significant findings, or report experimental measures instead of clinical outcomes.
- Findings from safety data based on RCTs and observational studies are consistent with known adverse events for medicinal cannabis and are typically mild to moderate, although these studies typically do not measure cannabis use disorder using validated screening tools or acute harms with higher THC concentration inhaled products.
- Only a small proportion of adverse events are being captured through traditional pharmacovigilance mechanisms.

## 1 Issues

**FIGURE 1:**
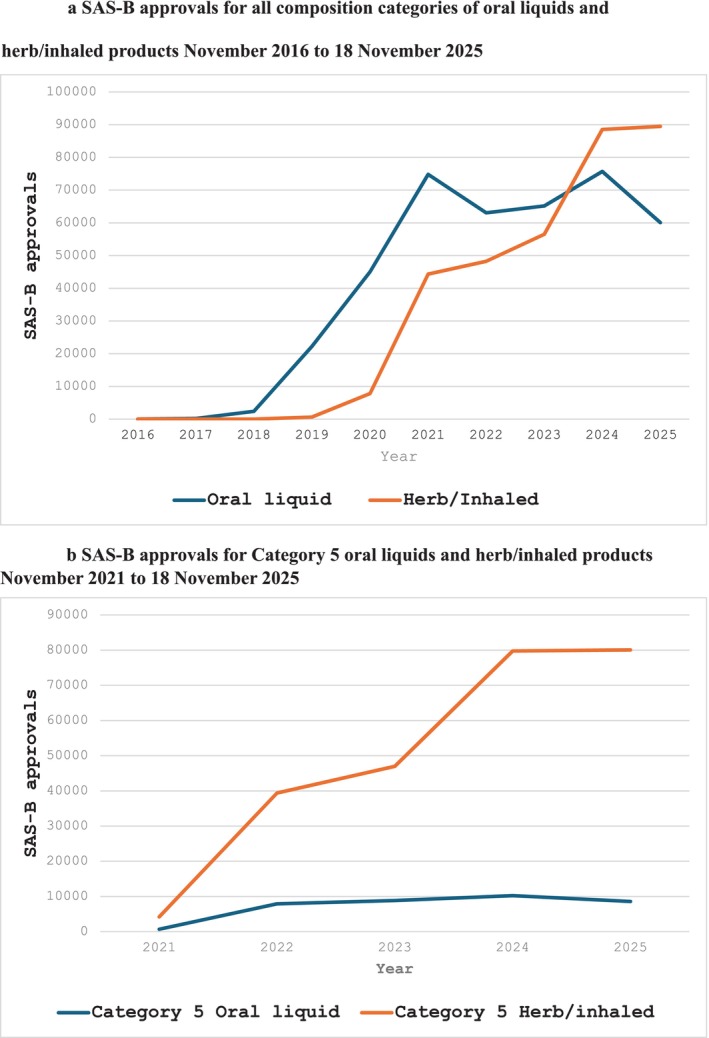
(a) SAS‐B approvals for all composition categories of oral liquids and herb/inhaled products November 2016 to 18 November 2025. (b) SAS‐B approvals for Category 5 oral liquids and herb/inhaled products November 2021 to 18 November 2025. Category 5 data became available from November 2021 onwards. (a) and (b) include aggregate data for oral liquids and inhaled dosage forms. Oral liquids dosage forms include oil, solution, liquid, tincture, oral liquid and spray solution dosage form categories. Herb/inhaled includes dried herb, inhalation, vaporisation, pressurised inhalation, concentrated extract, dry extract and powder for inhalation dosage form categories.

Higher delta‐9‐tetrahydrocannabinol (THC) concentration medicinal cannabis has increasingly become a topical issue, largely due to concerns about potential harms [1, 2]. In Australia, there have been anecdotal and published reports of harm attributed to Category 5 medicinal cannabis products, including psychosis and suicide [3, 4]. Medical professional associations and colleges have requested the removal of access to Category 5 medicinal cannabis products [5] and expressed concerns about prescribing patterns, direct to consumer marketing and telehealth clinic models [6, 7, 8]. Early in 2024, the Australian Health Professional Regulation Agency convened a national regulatory forum on medicinal cannabis [2], and the Therapeutic Goods Administration (TGA) is conducting a stakeholder consultation to inform regulatory reform [9]. These actions have occurred in response to transitions in the Australian medicinal cannabis market. Following the legalisation of medicinal cannabis in 2016, oral liquid products for oral or sublingual use were dominant in the legal Australian medicinal cannabis market (see Figure 1a). Between 2022 and December 2024, there was a shift from oral liquid‐based products (see Figure 1a) to higher THC concentration Category 5 medicinal cannabis dried flower (categorised by the TGA as ‘herb, dried’) and inhalation products with over 250,000 prescription application approvals (*n* = 250,425) (see Figure 1b) [10]. Since the incorporation of TGA medicinal cannabis categories in November 2021, prescription application approvals have covered a range of physical and mental health conditions, with 75.2% of Category 5 approvals for all dosage forms being for chronic pain (*n* = 126,955/299,601; 42.4%) and anxiety (*n* = 98,259/299,601; 32.8%). Based on 2025 data, Category 5 medicinal cannabis products accounted for half of all medicinal cannabis Special Access Scheme Category B (SAS‐B) approvals (*n* = 96,354/190,217).

Category 5 medicinal cannabis products are relatively heterogeneous, with multiple dosage forms and cannabinoid compositions. Most medicinal cannabis products are unregistered Schedule 8 Controlled Drugs, and Category 5 products are defined by the TGA as a proportion between cannabinoids being predominantly composed of THC or other cannabinoids > 98%, with cannabidiol (CBD) making up less than 2% of the total cannabinoid content. This product group represents a wide range of THC concentrations; though, depending on the quantity taken, it may not represent high THC doses. Category 5 products can also be devoid of THC and include other cannabinoids alone, such as cannabigerol (CBG) [11]. Some medicinal cannabis products available for inhalation in Australia have higher THC concentrations (> 30%) than would be anticipated to occur naturally in the plant [12]. THC extracts are another example of the higher THC concentration ranges of Category 5 products, reported to contain 75%–88% THC [11]. Four medical devices for vaporisation of medicinal cannabis have received TGA approval following assessment for quality, safety and performance. These include Mighty Medic, Mighty plus Medic, Volcano Medic 2 and SyqeAir Inhaler [13].

In international literature, there is no consistent definition of ‘high potency’ THC, with references to higher potency ranging from 10% to ≥ 60% THC, and multiple unit measures being used [14, 15]. There is a notable gap in the literature regarding health outcomes with the use of products that are consistent with the range of Category 5 products available through the Australian medicinal cannabis regulatory framework, particularly given the rapid increase in their use. This gap underscores the need for a comprehensive review to inform clinical practice and guide future research.

In response to recent concerns, proposals have been made to rapidly remove or restrict the entire Category 5 product group. Further to this, concerns have been raised about the need to consider dosage form and pharmacokinetics (e.g., oral liquid versus inhaled dosage forms), specific indications (e.g., palliative care), and if changes in access to Category 5 products are recommended to allow a sufficient timeframe to transition patients to other medicinal cannabis product categories, and to allow for THC dose titration to avoid withdrawal effects. However, despite these concerns and proposals, there has been limited evidence to enable a comprehensive understanding of the benefits and harms of this product group.

To address this gap, this review aimed to provide a concise overview of the evidence regarding the potential health effects of Category 5 medicinal cannabis products in adult populations. The primary research question guiding this review was ‘What are the health effects, including efficacy and safety, of Category 5 medicinal cannabis products in adults?’

## 2 Approach

A rapid review approach was required to deliver a synthesis of the literature within a short timeframe to inform policy decisions [16]. Alternate review methodologies (e.g., systematic and scoping) were carefully considered, and a rapid review approach was intentionally selected to meet deliverable timelines using the majority of rigorous systematic review methods to ensure transparency, reproducibility and a high level of evidence integrity. A study protocol was prospectively uploaded to the Open Science Framework https://doi.org/10.17605/OSF.IO/HNFUT. The review followed a structured approach, focusing on literature selection, data extraction and synthesis. Studies were selected based on predefined inclusion and exclusion criteria, focusing on adults using higher THC cannabis products that are consistent with the Category 5 product composition as defined by the TGA.

### 2.1 Eligibility Criteria

Eligible studies, including randomised controlled trials (RCT) and observational studies, were screened. Studies eligible for inclusion included those examining the efficacy (e.g., RCTs as the gold standard for examining causal relationships) and/or safety (e.g., RCTs and observational studies) of medicinal cannabis products consistent with the Category 5 composition as defined by the TGA for physical and/or mental health conditions in human adults (> 18 years old). Interventions consistent with Category 5 medicinal cannabis products, included extracts (75%–88% THC), dried flower (13% w/w (130 mg/g) to 60% THC), inhalation (800–880 mg/mL THC), inhalation pressurised (2.5–5 mg THC/actuation) and oral liquid (18 mg/mL to 100 mg/mL THC). The search was restricted to English‐language publications and covered the most recent 10 years to capture a substantial volume of literature.

Healthy volunteer (summarised in Leen et al. [17]) and naturalistic and app‐based data collection studies where cannabinoid composition could not be verified were excluded. Studies that aggregated data related to products with varying cannabinoid compositions (i.e., across TGA medicinal cannabis product categories) or where the original product concentration was altered were excluded, as reported health effects could not be attributed specifically to a product that would be equivalent to Category 5 cannabinoid composition. As the review progressed, there was an update to the public 6 month sponsor reporting data for medicinal cannabis products. In response, clarifications of the inclusion and exclusion criteria were made to determine where products were to be included or were determined to be out of the scope of the review. For transparency, on each occasion, the clarifications required were documented (see Table S1).

### 2.2 Search Strategy

A comprehensive search was conducted for the date range of 1 January 2014 to 30 July 2024, across multiple databases, including Medline, EMBASE, EMCARE, CINAHL, SCOPUS and the Cochrane Central Register of Controlled Trials, as well as grey literature sources. The full search strategy, including all search terms, was published prospectively via the Open Science Framework (https://doi.org/10.17605/OSF.IO/HNFUT). Additional articles were identified by screening the reference lists of retrieved studies.

### 2.3 Study Selection

Titles and abstracts retrieved from the search were screened independently in duplicate (MG, DA, NC, PK, AL and SN). Studies meeting the inclusion criteria based on title and abstract screening underwent duplicate full‐text review (MG, NC, DA and SN). Discrepancies between reviewers were resolved through discussion and, if necessary, consultation with a third reviewer.

### 2.4 Data Extraction

Data extraction followed a standardised process, capturing key details on study design, participant characteristics and interventions. Outcomes related to efficacy and safety were extracted for RCTs and safety outcomes were extracted for observational studies. Data were extracted using a standardised and pre‐piloted Microsoft Excel data extraction form. Single data extraction (DA, NC and DT) was completed, with fields including study details, focus, design, population, intervention, outcomes, effect measure, findings, conclusions, research gaps and limitations. Upon completion of data extraction, a second author (DA and MG) performed an independent cross‐check of all studies to ensure extraction accuracy.

### 2.5 Quality Assessment

During the initial rapid review, no formal quality assessment was performed due to the review's rapid nature, emphasising timely evidence synthesis [18]. The Newcastle–Ottawa Scale was employed to evaluate the methodological quality of the included observational studies [19]. Stars (*) were assigned for affirmative (‘Yes’) responses, with higher total scores reflecting better methodological quality. Studies receiving 7–9 stars were deemed high quality, those scoring 5–6 stars were classified as moderate quality and those scoring 0–4 stars were classified as poor quality. Randomised [20] controlled trials were assessed using the modified Jadad scale. Scores are based on the modified Jadad scale, assessing randomisation, appropriateness of randomisation, blinding, appropriateness of blinding, reporting of withdrawals/dropouts and allocation concealment. This scoring adjustment is reflected in total scores ranging from 0 to 7, with higher scores indicating better methodological quality. Quality categories are: High (5–7), Moderate (3–4), Low (≤ 2). Comprehensive details on the quality assessment tools and individual study scores are available in Tables S2 and S3. Consistent with rapid review methodology, assessments were independently conducted by two reviewers (DA and MG), and any scoring conflicts were resolved by discussion in a consensus meeting (DA, MG and SN).

### 2.6 Analysis

Results were synthesised narratively, highlighting key findings and trends across the included studies. For efficacy, RCT findings were grouped by physical and/or mental health condition. Where relevant, data subsets relating to products consistent with Category 5 medicinal cannabis products are reported. An enhancement to the narrative synthesis approach outlined in the protocol was applied during safety synthesis, incorporating Medical Dictionary for Regulatory Activities (MedDRA) terminology. Reported safety findings from RCTs and observational studies were grouped by adverse event severity, duration, study withdrawals and MedDRA terminology. Where appropriate, data were summarised in tables or figures to highlight key findings. Heterogeneous physical and/or mental health conditions, product compositions, dosage forms and routes of administration examined in the included studies precluded further meta‐analysis.

## 3 Key Findings

### 3.1 Study Selection

**FIGURE 2:**
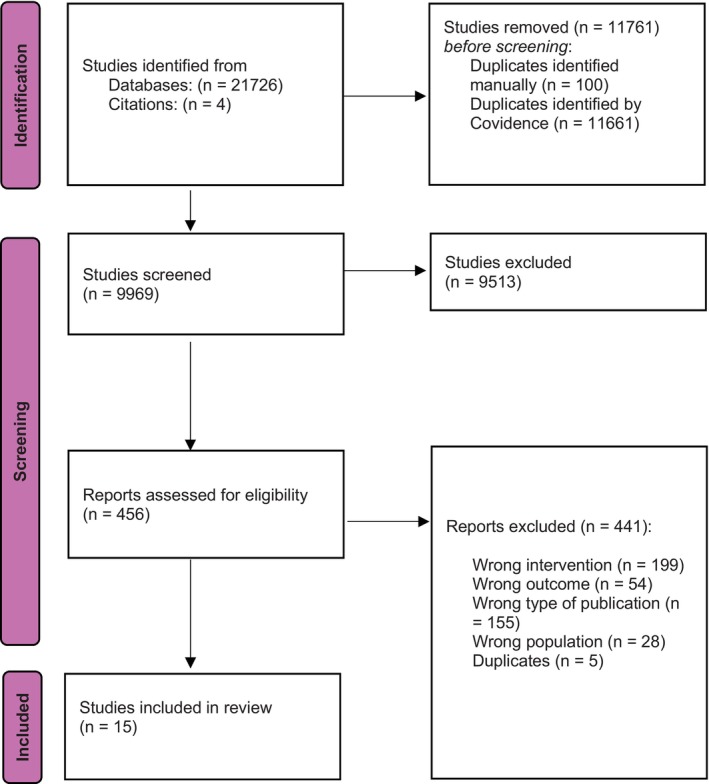
PRISMA flow diagram of the study selection process.

The study selection process was documented using the PRISMA flow diagram (see Figure 2).

The search identified 9969 records, of which 15 studies met the inclusion criteria. This comprised six RCTs and nine observational studies (see Tables A1 and A2). The highest THC concentration examined as part of the included studies was 22% w/w THC (220 mg/g THC), with THC concentrations ranging from 16% to 22%. Dosage forms and routes of administration in RCTs included oral or sublingual oil, and dried flower inhaled via metered dose inhaler (MDI), vaporisation or smoking. Observational studies reporting adverse events included a range of dosage forms, including dried flower products, oral liquids and capsules. A cannabis flower product with THC concentration ranging from 19.9% to 22% in included studies was examined in 55.6% (*n* = 5/9) of the observational studies, with three studies involving administration via MDI. For included studies where the same product brand name is specified and cannabinoid composition is not, the THC concentration of the product published on the company's webpage is used as the reference point (e.g., 22% THC) [21]. Seven studies were from Israel, two from Canada, two from the Netherlands, two from Australia, one from Italy and one from the United Kingdom. The conditions of interest included pain (e.g., occurring in Parkinson's disease, fibromyalgia and diabetes), anxiety, attention deficit hyperactivity disorder (ADHD), post‐traumatic stress disorder (PTSD), insomnia, multiple sclerosis, inflammatory bowel disease, advanced chronic obstructive pulmonary disease (COPD) and cancer‐related symptoms.

### 3.2 Quality Assessment

Of the 15 included studies, nine were observational and six were randomised trials. Overall, four RCTs were scored as high quality and two were moderate quality. Five observational studies were assessed to be low quality and four were moderate quality. Quality was assessed using different tools for observational and randomised studies; thresholds are not directly comparable (see Tables S2 and S3).

### 3.3 Narrative Synthesis: Higher THC Concentration Products and Physical and Mental Health Conditions

#### 3.3.1 Chronic Obstructive Pulmonary Disease

In a small RCT involving 16 participants with advanced COPD, a single inhaled dose of 35 mg of dried plant cannabis (18.2% THC, < 0.1% CBD) via vaporiser had no clinically meaningful effect on exertional breathlessness (intervention 2.7 ± 1.2 Borg units vs. placebo, 2.6 ± 1.3 Borg units (means ± SD)) and exercise endurance (intervention 3.8 ± 1.9 min vs. placebo, 4.2 ± 1.9 min) [22].

#### 3.3.2 Ulcerative Colitis

One eight‐week RCT (*n* = 32) examined the use of smoked dried cannabis flower (0.5 g equating to 80 mg THC) cigarettes (16% THC, 0.5% CBG, 0.1% CBD, < 0.1% of other cannabinoids) in patients with ulcerative colitis with mixed findings [23]. The study found a significant decrease in disease activity (10.9 (interquartile range 9–14) to 5 (interquartile range 1–7); *p* ≤ 0.000) and improvements in quality of life (QoL) (77 ± 4–98 ± 20; *p* = 0.000), general health, appetite, libido and concentration but no statistically significant improvement in endoscopic score or inflammatory markers.

#### 3.3.3 Pain

Four RCTs with 70 participants overall (*n* = 64 who received a Category 5 equivalent product) were identified that examined Category 5 equivalent products for pain [24, 25, 26, 27]. Product characteristics, routes of administration, outcome measures and study objectives differ substantially across the four RCTs, precluding further meta‐analysis.

A feasibility trial focused on pharmacokinetic, analgesic, cognitive and safety outcomes in participants with chronic neuropathic pain or complex regional pain syndrome (*n* = 27) found a significant dose dependent reduction in pain intensity with a single inhalation of dried flower (22% THC, < 0.1% CBD, < 0.2% cannabinol) via MDI [24]. Administered doses of 0.5 mg and 1 mg THC reduced mean maximum Visual Analogue Scale (VAS) pain score by 25.0% (1.95 points) and 39.4% (2.95 points), respectively. The VAS score reduction following the 1 mg dose was statistically significantly larger than placebo and 0.5 mg (*p* = 0.0015 [95% confidence interval 0.53, 2.23], *p* = 0.0058 [95% confidence interval 0.35, 2.08], respectively).

One experimental RCT assessed the effects of vaporised dried flower 100 mg equating to 22.4 mg THC and < 1 mg CBD (22% w/w THC (220 mg/g THC), < 1% w/w CBD) compared to products with other cannabinoid compositions and doses (described below) on measures of experimental pain in female patients with fibromyalgia (*n* = 20) [25]. Comparator cannabis doses and compositions included 200 mg of a dried flower product equating to 13.4 mg THC and 17.8 mg CBD (6.3% w/w THC, 8% w/w CBD (63 mg/g THC, 80 mg/g CBD)) and 200 mg of a dried flower product equating to < 1 mg THC and 18.4 mg CBD (< 1% w/w THC, 9% w/w CBD (90 mg/g CBD)). For THC containing products, there were inconsistent results across different pain measures with a significantly increased pressure pain threshold (i.e., higher pain tolerance) (*p* ≤ 0.01 for THC containing products and for the 22% w/w THC (220 mg/g), < 1% w/w CBD product alone 7 to 9 kgf (*p* = 0.006)), but no effect on spontaneous or electrical pain responses.

In a small Phase 1b trial (*n* = 8) in Parkinson's disease, three oral cannabinoid oil formulations were evaluated for tolerated dose and safety: a THC product (18.3 mg/mL THC, 0.2 mg/mL CBD), a balanced THC:CBD formulation (10:10; 9.8 mg/mL THC, 9.9 mg/mL CBD) and a CBD‐dominant formulation (1:20; 1 mg/mL THC, 20 mg/mL CBD). The mean maximum tolerated dose for the 18:0 product was 17.0 mg THC and 0.2 mg CBD daily [26].

In a small RCT (*n* = 15) of patients with chronic lumbar radicular pain, sublingual cannabis oil containing 20% THC (cannabinoid content information based on ClinicalTrials.gov ID NCT02560545) [28] in a dose of 0.2 mg/kg, equating to an average of 15.4 ± 2.2 mg THC, was used to examine functional brain changes associated with analgesia. Significant pain reduction was correlated with reduced connectivity between cognitive (e.g., thinking, knowledge acquisition and understanding) and affective (e.g., mood, emotions and feelings) and sensorimotor brain regions [27]. A significant reduction in subjective perceived ongoing pain was reported based on pre‐ and post‐THC administration VAS scores (*p* ≤ 0.005) and compared with placebo (*p* ≤ 0.05). Further research is warranted to determine if reduced functional connectivity of this nature impacts non‐pharmacological treatment outcomes.

### 3.4 Safety Assessment

Safety assessment included reported findings from RCTs and observational studies. Observational studies examined products consistent with Category 5 medicinal cannabis, across a broader range of physical and mental health conditions, including chronic pain, cancer‐related cachexia, anorexia and pain, multiple sclerosis, fibromyalgia, Crohn's disease, anxiety, ADHD, PTSD and insomnia.

#### 3.4.1 Adverse Event Severity and Duration

Most cannabis‐related adverse events were described as mild or moderate, with additional non‐standard language, including ‘minor’, ‘minimal’, ‘modest’ [22, 23, 24, 25, 26, 29, 30, 31, 32, 33, 34]. Serious adverse events were reported in an observational study of spontaneously reported events [29]. Moreover, adverse events were described as transient, reversible or resolved [24, 26, 29, 30, 31, 34, 35]. In a pilot study of patients with advanced cancer and cancer‐related cachexia and anorexia syndrome, psychoactive adverse effects that prevented physical activity occurred 1–2 h post‐administration of 5 mg (4.75 mg THC, 0.25 mg CBD) or 10 mg oral capsule doses, for a duration of 2–3 h. Dose reductions were implemented if adverse events occurred [32]. In an RCT examining dose tolerance and safety of cannabis formulations for pain in Parkinson's disease, adverse event resolution (e.g., dizziness) was reported following dose reduction of THC:CBD oral oil 18:0 (18.3 mg/mL THC, 0.2 mg/mL CBD) [26]. In a retrospective study (*n* = 143) of participants with mainly chronic neuropathic pain (*n* = 103/143; 72.0%) taking granulated cannabis flowers (22% THC) via MDI, 33.6% (*n* = 48) of patients experienced adverse events during the titration phase compared to ≤ 4% of patients at 3–15 months. The average post‐titration dose was 1.5 mg THC ± 0.7 mg THC. Notably, cannabis naïve patients were commenced on a lower starting dose than participants with previous experience [35].

#### 3.4.2 Study Withdrawals and Lack of Effectiveness

In participants taking a 10 mg (9.5 mg THC, 0.5 mg CBD) oral capsule dose as part of a pilot study for anorexia and cachexia symptoms in advanced cancer, three participants withdrew due to mild to moderate adverse effects, including tiredness, dizziness, disorientation, anxiety, hallucinations and alterations in general functioning. Thereafter, a reduced dose of 5 mg (4.75 mg THC, 0.25 mg CBD) was taken by the remaining cohort to counter adverse events. Twenty‐three percent (*n* = 3/13) of participants taking 5 mg capsules withdrew from the study due to a similar adverse event profile. During the first fortnight of treatment, four withdrawals were attributed to cannabis adverse events, and two participants withdrew due to chemotherapy‐related adverse events and disease progression. Thereafter, two patient withdrawals were also attributed to cannabis adverse events and three were due to disease progression [32].

Four additional studies reported ineffectiveness and/or discontinuation of participation due to cannabis‐related adverse events. In an RCT examining cannabis flowers with different cannabinoid compositions (including a product with 22% THC (220 mg/g THC), < 1% CBD inhaled via vaporiser) in a population with fibromyalgia, three out of five study withdrawals were due to nausea [25]. In an observational study of granulated cannabis flowers (22% THC) inhaled via MDI in a cohort with mainly chronic neuropathic pain (*n* = 103/143; 72.0%), adverse events (*n* = 3/143; 2.1%) and ineffectiveness (*n* = 5/143; 3.5%) were recorded as reasons for study withdrawal [35]. Lack of efficacy was reported as an adverse event for one participant taking a cannabis flower product 22% THC orally in an observational study of spontaneously reported adverse events [29]. In an observational study in patients with chronic pain, anxiety, PTSD and multiple sclerosis, two participants experienced increased anxiety, with one taking a vaporised cannabis flower product (22% w/w THC, < 1% w/w CBD), ceasing study participation. The second participant, who was taking a cannabis flower product (20% w/w THC, < 1% w/w CBD) via vaporiser, continued medicinal cannabis following the prescription of different cannabinoid formulations [33].

#### 3.4.3 Difficulty Stopping Use and Behavioural Consequences

In an RCT of patients with ulcerative colitis, difficulty in stopping use (*n* = 5/17; 29.4% compared to *n* = 2 in the placebo group) was reported with 0.5 g of cannabis flower in the form of cigarettes (16% THC, 0.5% CBG, 0.1% CBD, < 0.1% of other cannabinoids) containing 80 mg of THC. Fifteen participants elected not to be involved with post‐RCT follow‐up, with reasons including a ‘wish to stop cannabis’ (*n* = 7) [23]. In an Australian observational study, a Cannabis Based Medicines Questionnaire was used to collect data on the behavioural consequences of medicinal cannabis use, although questionnaire outcomes are not detailed [33].

#### 3.4.4 Cough

Cough was reported in six studies (four RCTs and two observational studies) that included inhalation of dried cannabis herb or flower [22, 23, 24, 25, 31, 35], including one study that classified cough as miscellaneous instead of a respiratory disorder [35]. A post‐inhalation cough was experienced by six out of 16 participants with advanced COPD following single dose inhalation via vaporiser of 35 mg of dried herb cannabis (18.2% THC, < 0.1% CBD) in an RCT that examined effects on exertional breathlessness and exercise endurance [22]. In addition to the reported absence of a clinically meaningful effect on exertional breathlessness, clinically significant worsening of exertional breathlessness was reported by five of the six participants with post‐inhalation cough [22]. In a separate RCT involving patients with ulcerative colitis, cough (*n* = 7/17; 41.2%) was the most frequently reported adverse event following use of 0.5 g of cannabis flower in cigarette form (16% THC, 0.5% CBG, 0.1% CBD, < 0.1% of other cannabinoids) [23]. Cough was reported as an adverse event in two observational studies (*n* = 3/21, median dose per day 1.5 mg THC; and *n* = 4/143, average post‐titration dose 1.5 mg THC, respectively) [31, 35] and one RCT (single dose 0.5 mg THC: *n* = 4/22, number of reports *n* = 6/60; 1.0 mg THC: *n* = 11/20, number of reports *n* = 13/66) where cannabis flower THC 22% was inhaled via MDI [24]. Over two‐thirds of participants experienced a cough (*n* = 14/20; 70.0%) with a 22.4 mg THC dose using 100 mg vaporised cannabis flower (22% THC (220 mg/g), < 1% CBD) in an RCT investigating analgesic effects in fibromyalgia [25].

#### 3.4.5 Dizziness, Headache, Lightheadedness and Somnolence

Dizziness, headache, lightheadedness and/or somnolence were reported in 11 studies. Dizziness was reported in nine studies [23, 24, 25, 26, 29, 32, 35, 36, 37], including one study that reported dizziness as an ear and labyrinth disorder [29] and two studies that classified dizziness as a nervous system disorder [35, 36]. Headache was reported in four studies [25, 29, 30, 35] and light‐headedness in two studies [26, 34]. In a small (*n* = 8) Phase 1a, open label study of cannabis administered via MDI (15.1 ± 0.1 mg cannabis flower 19.9% THC, consistent with 3.1 mg ± 0.02 mg THC) in participants with chronic neuropathic pain, *n* = 7 participants experienced light‐headedness [34]. Sleepiness, somnolence or drowsiness were reported in five studies [24, 25, 29, 35, 36]. Two of these studies classified this as a nervous system disorder [29, 35] and one as a psychiatric disorder [36]. Due to the diversity in populations, dosage forms and routes of administration, detailed information on each study is available in Tables A1 and A2.

It is important to consider participant baseline characteristics, including their previous experience with cannabis, when interpreting study outcomes. In an observational registry study describing the effects of vaporised cannabis flower (20% w/w THC, < 1% w/w CBD), a mild headache was reported for one participant who had previous experience with cannabis and transient memory loss was reported for a cannabis naïve patient. The small proportion of adverse events in this study was attributed to 95.6% (*n* = 329/344) of participants having previous experience with cannabis [30].

#### 3.4.6 Anxiety

Four observational studies examined the effectiveness of Category 5 equivalent products for various physical health conditions and/or mental health conditions, including anxiety and PTSD [30, 33, 36, 37] (see Table A2 for population‐specific characteristics). Anxiety was reported as an adverse event in six observational studies [29, 32, 33, 35, 36, 37]. An RCT examining a 35 mg single dose inhalation of dried herb cannabis (18.2% THC, < 0.1% CBD) by vaporiser on COPD symptoms reported a significant decrease in anxiety (pre‐treatment 17.6 ± 18.0, post‐treatment 8.2 ± 1.2; *p* ≤ 0.05), and another experimental brain imaging study in chronic neuropathic pain that utilised 20% THC sublingual oil (average THC dose 15.4 ± 2.2 mg) reported no significant change compared to placebo, although this was not a primary outcome measure or objective of either study [22, 27]. A lack of a significant association between THC dose and anxiety was reported in an observational study [36].

For studies involving multiple product compositions, some reported variance in adverse event frequency, while others reported no difference. One observational study examined cannabis flower (19% THC, < 1% CBD) and other product compositions (12% THC, < 1% CBD; 6% THC, 7.5% CBD) for a range of health conditions, including chronic pain and multiple sclerosis [37]. The products were predominantly inhaled (81.4%; *n* = 83/102) or used as a tea. A higher level of dejection (*p* = 0.02) and anxiety (*p* = 0.006) was reported with the 19% THC cannabis flower product compared with another product containing a lower THC concentration (6% THC, 7.5% CBD). The average dose did not differ between the various cannabinoid compositions [37]. In an RCT investigating analgesic effects on fibromyalgia pain, there were no statistically significant differences (*p* ≥ 0.05) in adverse event frequency between different cannabis flower product compositions (< 1% w/w THC, 9% w/w CBD (90 mg/g CBD); 22% w/w THC (220 mg/g THC), < 1% w/w CBD; 6.3% w/w THC, 8% w/w CBD (63 mg/g THC, 80 mg/g CBD)) [25].

An Australian observational study examining participant health outcomes with a variety of oral liquid and capsule cannabis products in anxiety and PTSD reported adverse events by cannabinoid composition. For THC only products, reported psychiatric disorders (total adverse events *n* = 28/71; 39.4%, and number of participants reporting an adverse event *n* = 12/19; 63.2%), included somnolence (classified as a psychiatric disorder in this study), anxiety, depression and euphoria. Euphoria was reported by a greater proportion of the THC only group (*n* = 3/19 participants; 15.8%) compared to the THC dominant group (*n* = 5/51; 9.8%). Participants taking CBD only products most commonly reported somnolence (*n* = 88/297; 29.6%) and dry mouth (e.g., gastrointestinal disorder) (*n* = 87/297; 29.3%). A greater proportion of the CBD only group did not report adverse events compared to other product formulations (*n* = 120/297; 40.4%) [36]. Another observational study in participants with chronic pain, anxiety, PTSD and multiple sclerosis reported that two patients (out of 278 taking a variety of cannabinoid compositions and dosage forms) experienced anxiety following the use of THC only flower products [33].

#### 3.4.7 Confusion and Restlessness

Confusion [23, 29, 35, 36] and restlessness [23, 24, 26, 35] were reported in four studies involving oral or inhaled (e.g., smoked, MDI) routes of administration. One out of the four studies reported confusion as a nervous system disorder [35] and two studies reported confusion as a psychiatric disorder [29, 36].

#### 3.4.8 Drug High

In line with expected psychoactive effects following use of THC, drug high was reported as a subjective or adverse effect in three RCTs [22, 24, 25]. In one of the RCTs that included participants with COPD, a statistically significant increase in drug high rating (pre‐treatment 1.9 ± 2.1 and post‐treatment 4.8 ± 4.5 (mean ± SD), *p* ≤ 0.05) was reported post‐inhalation of dried herb cannabis (18.2% THC, < 0.1% CBD) via vaporiser [22]. In an RCT evaluating cannabis flower (22% THC, < 0.1% CBD) via MDI for chronic pain, drug high was the most commonly reported adverse event. A dose dependent effect was also reported with a doubling of drug high intensity with the 1 mg dose compared to the 0.5 mg dose (0.5 mg: *n* = 12/22 participants and *n* = 13/60 reports; 1.0 mg: *n* = 16/20 participants and *n* = 16/66 reports) [24]. In a separate RCT that included a cohort of patients with fibromyalgia and chronic pain, following the use of cannabis flower products (22% w/w THC (220 mg/g THC), < 1% w/w CBD or 6.3% w/w THC and 8% w/w CBD (63 mg/g THC, 80 mg/g CBD)) via vaporiser, most participants experienced a drug high (both *n* = 16/20; 80.0%, respectively) compared to placebo (*n* = 2/20; 10.0%) or a low THC (9% w/w CBD (90 mg/g) and < 1% w/w THC) cannabis flower product (*n* = 8/20; 40.0%). The drug high was reported to be disliked by most participants. The intensity rating for drug high was lower with 200 mg of a low THC cannabis flower product (< 1% w/w THC, 9% CBD w/w) than with 100 mg of a 22% w/w THC (containing 22.4 mg THC) and < 1% w/w CBD product (*p* = 0.003) or 200 mg of a more balanced cannabinoid ratio product (6.3% w/w THC, 8% w/w CBD equating to 13.4 mg THC) (*p* ≤ 0.001). A strong correlation was reported between the magnitude of drug high and spontaneous pain scores (ρ = −0.5, *p* < 0.001) [25].

#### 3.4.9 Gastrointestinal

Gastrointestinal disorder adverse effects were reported in six studies [24, 25, 26, 29, 35, 36], involving inhaled (e.g., MDI and vaporiser) and oral (e.g., liquid and capsule) dosage forms in different physical and/or mental health conditions (e.g., chronic pain, anxiety and PTSD). Nausea was reported as an adverse event in six studies [24, 25, 26, 29, 35, 36] and dry mouth in four studies across a range of cannabinoid compositions and oral and inhaled dosage forms [24, 29, 35, 36] (see Tables A1 and A2 for detailed information on each study). In an observational study involving THC‐only oral liquid or capsules for anxiety and PTSD, the association between THC concentration and gastrointestinal adverse events was not likely to be clinically significant (nausea OR = 1.008, *p* = 0.008 and dry mouth OR = 1.010, *p* = 0.005) [36].

#### 3.4.10 Cardiovascular

No significant change in cardiovascular measures, including heart rate and blood pressure, was reported in an RCT involving chronic neuropathic pain and a single 20% THC sublingual dose (average dose 15.4 ± 2.2 mg THC) [27]. Palpitations were reported in one observational study of cannabis flower (22% THC) administered via MDI (*n* = 2/143; 1.4%) [35]. Arrhythmias (*n* = 4) were reported following the use of a cannabis flower product (22% THC) taken orally in an observational study of spontaneously reported adverse events [29].

## 4 Implications

The scope of this review focused on a specific set of products (i.e., products consistent with Category 5, also known as THC only) relevant to the Australian medicinal cannabis regulatory context. The upper limit of THC concentration in the retrieved RCTs and observational studies was 22% w/w (220 mg/g), with THC concentration ranging from 16% to 22%.

The rapid review identified a small number of RCTs (*n* = 6) that report on the potential efficacy of products consistent with Category 5 composition in adults in a limited range of physical and mental health conditions. Three of the four RCTs involving populations with pain examined tolerated dose and safety [26], experimental pain measures [25] and functional brain changes [27], respectively, and were not designed to examine clinical effects on pain. One RCT reported a clinically meaningful (≥ 30%) reduction in chronic neuropathic pain or complex regional pain syndrome with a single 1 mg THC dose via MDI, but did not examine longer term outcomes [24]. Mixed outcomes (e.g., symptomatic relief vs. endoscopic findings) were reported in a study of smoked dried cannabis flower in ulcerative colitis [23]. In an RCT involving vaporised dried herb cannabis in COPD, a lack of clinically meaningful effects was reported for primary respiratory outcomes [22].

In addition to the mixed outcomes reported, the ability to draw meaningful conclusions about efficacy was limited due to some studies involving only single dose administration or including limited follow up periods, resulting in evidence gaps related to the long‐term efficacy of Category 5 equivalent products. The efficacy results of single dose studies are unlikely to represent the effects seen with repeated administration, as repeated administration can lead to tolerance. For some common indications, such as anxiety, no RCTs examining efficacy with higher THC concentration Category 5 medicinal cannabis products were retrieved that met study inclusion criteria. Systematic reviews of RCT studies for anxiety include CBD‐based products [38] and are outside of the scope of the rapid review, and included studies involving products containing THC were observational studies [30, 33, 36].

Overall, reported adverse events from RCTs were mild to moderate and included dizziness, cough, drug high and nausea. One study in people with ulcerative colitis reported ‘difficulty to stop use’ of cannabis for five out of seventeen participants, highlighting the need to incorporate methodological approaches that facilitate robust assessment of and reporting on the prevalence of cannabis use disorder (CUD) [23]. From a clinical perspective, if a patient is commenced on medicinal cannabis, regular clinical monitoring of symptom improvement against treatment goals, adverse effects, mental health status, potential drug interactions and overall functioning is recommended.

The clinical effects and potential for adverse events with medicinal cannabis depend on the total dose of THC administered, not just concentration in isolation. Of note, the definition of Category 5 products means a broad range of products are captured. Product safety profiles may differ greatly, for example, with smaller doses of THC (e.g., 0.25 mg, 0.5 mg or 1 mg doses) found in studies in this review, compared with larger doses of products containing up to 88% THC being prescribed in practice. Some published studies on the health effects of higher THC concentration products suggest that these allow for lower doses to be taken. Yet, evidence on whether study participants effectively self‐titrate doses with higher THC concentration products is mixed; reduced inhalation volumes with stronger products have been reported, although this may not fully offset the higher THC exposure [39, 40, 41].

While cannabis use patterns and health outcomes have been explored in the context of nonmedical use, an evidence gap exists related to higher THC concentration medicinal cannabis use patterns. THC concentration needs to be considered alongside dose, dosage form, route of administration, duration, quantity and frequency of use. To date, there is limited research documenting actual medicinal cannabis consumption patterns with higher THC inhaled products (e.g., documenting standard doses used for different indications or capturing administration frequency). Studies using Australian medicinal cannabis use patterns (such as those that use prescribing or dispensing data) may help to address this gap, to understand whether actual use patterns go beyond doses tested in research studies.

Reported adverse events in observational studies were consistent with known adverse events for THC, including psychiatric (e.g., anxiety), nervous system (e.g., dizziness) and gastrointestinal effects (e.g., nausea). While increased appetite and euphoria are sometimes reported as adverse effects, these may be desirable effects [32, 36]. Serious adverse events were reported in an observational study of spontaneously reported adverse events, although most were mild to moderate across all observational studies. Nevertheless, in some conditions, such as cancer‐related cachexia and anorexia, withdrawal rates from Category 5 equivalent products due to adverse events highlight tolerability considerations for vulnerable populations [32].

Safety considerations with medicinal cannabis, based on published studies, are included in medicinal cannabis guidance documents. The TGA medicinal cannabis guidance documents note that, regardless of cannabinoid composition category, medicinal cannabis is not a first‐line treatment strategy for any physical or mental health condition and may be considered on a case‐by‐case basis when all standard therapeutic approaches have been trialled or have been deemed inappropriate due to adverse effects or contraindications. Australian and international condition‐specific clinical guidance has previously been published, although it does not necessarily include information for clinicians on considerations for Category 5 medicines [42]. Unfortunately, there is a lack of definitive efficacy data from identified studies (i.e., few or no adequately powered definitive studies with Category 5 products that would enable such guidance). As with other recent systematic reviews [43], the findings of this review continue to identify that widespread use of Category 5 medicinal cannabis products is not well aligned with existing evidence. While safety considerations with higher THC medicinal cannabis products have been added to select guidance (e.g., the Royal Australian and New Zealand College of Psychiatrists Clinical Memorandum on the therapeutic use of medicinal cannabis products) [44], there remains a gap in the integration across existing health professional resources. Furthermore, there is a clear need for public health education for patients.

A critical evidence gap is the lack of any research into the potential efficacy and safety considerations related to products (i.e., consistent with TGA Category 5 products) with THC concentrations greater than 22% w/w (220 mg/g), which appear to be increasingly represented in prescription application approvals in Australia for anxiety and chronic pain [10, 11]. The absence of RCTs involving products consistent with Category 5 for anxiety is notable, as it is the second most common indication for Category 5 prescription application approvals in Australia [10]. Similarly, as some outcome measures were indirect (e.g., experimental pain rather than clinical outcomes), limited conclusions can be drawn relating to clinical efficacy.

There have been Australian case reports of anxiety, psychosis and cannabis hyperemesis following the prescription of Category 5 medicinal cannabis products [45, 46]. Concerns about acute harms resulting from the use of higher THC concentration medicinal cannabis products were a key factor that triggered this rapid review. Apart from publication types (e.g., case studies and Letters to the Editor) that were excluded, there were limited Australian studies that examined acute presentations related to psychosis and higher THC concentration medicinal cannabis [4, 45]. Even where adverse events are documented, the product composition leading to harm is not always clear [4].

Five of the included studies were single dose studies [22, 24, 25, 27, 34], and no study routinely measured or clearly reported detailed outcomes related to the development of CUD using published, validated screening tools. Therefore, limited conclusions can be made about the clinical relevance for chronic conditions and longer term adverse events despite them being identified as a concern with higher THC concentration products in the broader literature. This is further highlighted by the findings of a German pain management centre cross‐sectional study that suggested both overestimation and underestimation of CUD in patients with chronic pain taking prescribed medicinal cannabis. This study also included the prescription of cannabis flower (*n* = 20/187; 10.7%), including products containing 22% THC and < 2% CBD, although small sample sizes precluded formulation subgroup analyses [47]. This is a notable gap, given that an international systematic review estimated rates of CUD among people who report cannabis use for therapeutic purposes to be 25% (95% confidence interval 18%, 33%) [48]. A study by Kritikos et al. [49] reported a lack of significant difference in CUD between past month medical and nonmedical use, although emphasised the importance of incorporating quantity and frequency metrics. Several other characteristics may confer a higher risk of CUD, including younger age, male, inhaled cannabis use and mental health or chronic non‐cancer pain conditions [48]. The application of CUD criteria in the context of prescribed medicinal use, particularly tolerance and withdrawal, and the overlap between medical and nonmedical use, requires further research.

To date, few studies have mapped the changes in prevalence of adverse events at the population level with increasing use of higher THC concentration products, identifying an important gap in our current understanding of these harms. A systematic review of high‐potency cannabis observational and experimental studies by Lake et al. [14] emphasised the need for prospective studies to investigate therapeutic, cardiovascular, respiratory, pre‐ and peri‐natal and cancer outcomes. Lake et al. [14] found a relatively consistent association between indicators of ‘problem’ cannabis use and higher‐potency cannabis but noted the low quality and certainty in existing evidence. In a separate systematic review focused on mental health outcomes, a broader range of study types were represented, including RCTs, interventional studies and observational studies. The authors concluded that higher THC concentration products (e.g., defined as > 10% or > 5 mg THC/serving, or labelling indicating a higher potency concentrate dosage form) were associated with adverse mental health outcomes for psychosis, schizophrenia and CUD [43]. Scoping reviews and systematic reviews of RCTs and observational studies are available relating to higher THC concentration products [14, 43, 50], although the products included for the most part do not have a cannabinoid composition that is consistent with Australian Category 5 products, or the composition cannot be verified, limiting the application of these reviews to the Australian context.

### 4.1 Limitations

Rapid reviews are subject to inherent limitations to enable rapid data capture, synthesis and reporting. The heterogeneity in Category 5 products, studies and outcome measures limits the ability to draw strong conclusions. The majority of studies of higher THC concentration products did not meet the inclusion criteria due to an inability to verify the cannabinoid profile of the products to determine if they were consistent with Category 5 composition or due to aggregation of data related to multiple product compositions (e.g., TGA medicinal cannabis categories). As a result, the evidence base consisted of a relatively smaller number of eligible studies.

Within Category 5 product composition definitions, there is also considerable heterogeneity in product cannabinoid concentrations, dosage forms, routes of administration (e.g., oral versus inhaled) and associated pharmacokinetic variability, doses and dosing (e.g., single vs. repeated dosing and set dosing versus individualised self‐titration). Furthermore, there can be substantial variability in inhalation technique for vaporised products, and oral products are subject to variance in bioavailability. Our findings show an absence of evidence related specifically to products with cannabinoid composition consistent with Category 5 products, particularly at the higher end of THC concentration (e.g., 30%–80% THC or higher), which represents a notable gap given the increasing prescription approvals for Category 5 products in Australia.

There are also limitations in the quality of the studies and the conclusions that can be drawn based on the study designs. Nine of the 15 included studies had small cohorts (*n* ≤ 50 participants) [51] and, as such, further higher quality research is needed in larger cohorts over a longer timeframe to enable robust conclusions on safety to be made. Small samples may not be adequately powered to detect intervention effects. Although four of the six RCTs met criteria for ‘high’ quality, challenges in participant blinding were described in *n* = 4/5 of the included placebo‐controlled studies, including a high proportion of participants who correctly identified which intervention they had received in two studies (*n* = 13/20; 65.0% and *n* = 12/16; 75.0%, respectively) [22, 25] and incomplete or absent post‐intervention data about participant intervention identification in two additional studies [23, 24]. This is notable given the potential bias introduced by functional unblinding and expectancy effects with cannabinoids [52, 53]. When considered alongside study quality limitations, this limits generalisations about the magnitude of the intervention's effects (i.e., products consistent with Category 5). The included studies involve a limited number of clinical indications and specific populations. Due to a lack of visibility around product usage in Australia, and for what specific indications, it is challenging to know how the results of the existing research may be translated to current use in Australia. Limitations of spontaneous adverse event databases have been previously reported and include incomplete data and under‐reporting. In light of these considerations, we acknowledge that while our review provides valuable insights, it should be interpreted with an understanding of these limitations.

Future research could examine the proportion of individuals prescribed Category 5 products who are also prescribed medicinal cannabis products from other categories, as this would provide insights into the overall patient dose of different cannabinoids. Moreover, comprehensive pharmacovigilance data capture, including real world data, is needed to better understand adverse event rates in relation to the number of prescriptions dispensed in the population. Pharmacokinetic and dose–response and ‐trajectory data could be examined in further research, taking into account the diverse range of cannabinoid compositions, dosage forms, routes of administration and physical and/or mental health conditions for which medicinal cannabis prescription approvals are being issued.

## 5 Conclusion

Overall, the rapid review found a small number of RCTs and observational studies that examined the potential efficacy and safety of Category 5 products with THC concentrations of up to 22% w/w THC (220 mg/g THC) in a limited range of physical and mental health conditions. There is mixed evidence of efficacy that is specific to health condition and medicinal cannabis product. The safety profile of lower THC concentration Category 5 medicinal cannabis products is consistent with known adverse effects to THC, noting that the THC concentrations examined in included RCTs and observational studies are considerably lower than the upper limit of THC potencies for Category 5 products (i.e., up to 88% in Category 5 products at the time of the review) and may not be generalisable across all Category 5 products. As such, the use of many higher THC concentration Category 5 medicinal cannabis products is not supported by the existing evidence base.

## Author Contributions

**Myfanwy Graham:** conceptualisation, design, protocol development, literature searching, screening, data extraction, data synthesis, visualisation, writing review and editing. **Dereje Assefa:** screening, data extraction, writing review and editing. **Ngo Cong‐Lem:** screening, data extraction, writing review and editing. **Suzanne Nielsen:** conceptualisation, design, protocol development, screening, writing review and editing, supervision.

## Funding

This work was supported by the Australian Therapeutic Goods Administration, Department of Health, Disability and Ageing, Australian Government. M.G is the recipient of an NHMRC Postgraduate Scholarship (GNT#2030765) and Monash Graduate Research Excellence Scholarship. SN is the recipient of an NHMRC Leadership Fellowship (GNT#2025894). The funder(s) had no role in the design of the study, data retrieval and synthesis, preparation of the manuscript, or the decision to publish.

## Conflicts of Interest

M.G is an appointed member of the Therapeutic Goods Administration's Medicinal Cannabis Expert Working Group. This article does not represent the views of the TGA or the Expert Working Group. All other authors report no conflicts of interest.

## Supporting information


**Table S1:** Inclusion and exclusion criteria.

**Table S2:** Newcastle–Ottawa Scale quality assessment of included observational studies.
**Table S3:** Modified Jadad scores of the included randomised clinical trial studies.

## Data Availability

The data that support the findings of this study are available from the corresponding author upon reasonable request.

## Appendix A

**TABLE A1:** Randomised controlled trials—Efficacy and safety (*n* = 6).

Citation	Aims or objectives	Study design	Location and sample characteristics	Cannabis product consistent with Category 5*	Comparator (or comparison products)	Main findings (focused on cannabis products consistent with Category 5)
Abdallah 2018	To test the hypothesis that inhaled vaporised cannabis improves exercise endurance and reduces exertional breathlessness in individuals with COPD.	RCT, crossover	Canada *n* = 16; M 10, F 6; Mean age (±SD) 65.4 ± 7.7 years Individuals with advanced COPD	NCT03060993 18.2% THC, < 0.1% CBD Dried herb cannabis; Inhalation, via vaporiser; 35 mg cannabis Single dose inhalation	Placebo (0.3% THC, < 1.0% CBD), although 12 of the 16 participants correctly identified intervention versus placebo visits.	The authors concluded that single dose inhalation of vaporised cannabis had no clinically meaningful positive or negative effect on airway function, exertional breathlessness and exercise endurance in adults with advanced COPD. However, six participants exhibited post‐cannabis inhalation cough and clinically significant worse exertional breathlessness was reported by five participants. A participant was excluded from the study due to an adverse event (unrelated to the study intervention) and was not included in the analysis. Modest and statistically significant effects included reduced anxiety and increased feeling of being ‘drunk’, ‘high’, ‘stoned’, positive and negative effects and liking drug effects. Funding: Storz & Bickel America Inc. supplied vaporiser.
Almog 2020	To investigate the pharmacokinetics, analgesic effects, cognitive performance, and safety of delta‐9‐THC inhaled via a medical device in patients with chronic pain.	RCT, crossover	Israel *n* = 27; M 19, F 8; Mean age (±SD) 48.3 ± 11.9 years Chronic focal or distal symmetrical (diabetic) neuropathic pain (*n* = 21), complex regional pain syndrome (*n* = 6)	22% THC, < 0.1% CBD, < 0.2% CBN. Dried flower; Inhalation via MDI; 0.5 mg or 1 mg THC Single dose inhalation	Placebo (< 0.01% cannabinoid compounds)	Both doses, 0.5 mg and 1 mg THC, but not the placebo showed a dose‐dependent pain intensity reduction (e.g., mean maximum reduction in Visual Analogue Scale pain score 25.0% and 39.4%, respectively). A statistically significant and clinically meaningful (≥ 30%) reduction was reported for the 1 mg dose. Adverse events: 0.5 mg *n* = 22 patients (*n* = 60 reports) and 1 mg *n* = 20 patients (*n* = 66 reports). *n* = 4 Serious adverse events (SAE) (unrelated to study intervention); *n* = 1 withdrawal due to dizziness during a cognitive test. Study intervention related adverse events (80%^) and mostly mild (95%^). Common adverse events included ‘high’ (20.3%), cough (10.1%), weakness (9.2%), restlessness (8.2%), dry mouth (7.2%), dizziness (6.8%), sleepiness (6.3%), nausea (4.8%) and a moderate reduction in blood pressure (3.9%). No consistent impairments in objective cognitive performance assessment were evident. The intensity of drug high was double with 1 mg compared to 0.5 mg dose. Pharmacokinetic data is not reported here. Funding: Syqe Medical Ltd.
Di Luca 2023	To determine the maximum tolerated dose and safety of delta‐9‐THC and CBD formulations for pain management in patients with Parkinson's disease.	Randomised trial, Phase 1b	Canada *n* = 8; M 2, F 6; (*n* = 2 in the THC only group; M1, F1); Mean age (SD) in THC only group 52.5 (4.9) years Pain in Parkinson's disease	NCT03639064 THC:CBD oral oil 18:0 (18.3 mg/mL THC, 0.2 mg/mL CBD) Mean maximum tolerated dose (SD) 0.9 mL/d (0.1) for the THC only group (17.0 mg THC and 0.2 mg CBD); maintenance period 35–42 days	THC:CBD 10:10 (9.8 mg/mL THC, 9.9 mg/mL CBD) 1:20 (1 mg/mL THC, 20 mg/mL CBD)	Adverse events in the THC‐only group (all *n* = 1) included light headedness, dizziness, nausea, body paraesthesia and restlessness. No serious adverse events or study withdrawals reported, and adverse events (e.g., dizziness) resolved following dose reduction. Incomplete recruitment and early study termination due to investigational product expiry. Funding: Parkinson Canada Pilot Project Grant.
Naftali 2021	To investigate the clinical, laboratory and endoscopic effects of medical cannabis in patients with mild to moderate ulcerative colitis.	RCT	Israel *n* = 32; M 18, F 14; Mean age 30 years Ulcerative colitis	NCT01040910 (16% THC, 0.5% CBG, 0.1% CBD), < 0.1% of cannabichromene, cannabidivarin and delta‐8‐THC Inhalation, smoked cigarettes; 0.5 g dried cannabis flowers (80 mg THC) twice daily 8 weeks +2 week follow up (*n* = 17 patients were followed up in clinic over 12 months)	Placebo cigarettes (< 0.4% THC)	Significant reduction in Lichtiger disease activity index score. QoL, general health, appetite, libido and concentration improvement. No statistically significant improvement in endoscopic score or inflammatory markers (e.g., C‐reactive protein and calprotectin). Adverse effects in the THC group included cough (*n* = 7/17; 41.2%), dizziness (*n* = 6/17; 35.3%), confusion (*n* = 5/17; 29.4%), difficulty stopping cannabis use (*n* = 5; 29.4% compared to placebo *n* = 2, *p* = 0.543), behavioural change (*n* = 4/17; 23.5%), restlessness (*n* = 2/17; 11.8%), shortness of breath (*n* = 1/17; 5.9%). Fifteen participants did not continue follow‐up, with reasons including a ‘wish to stop cannabis’ (*n* = 7). Four patients discontinued use due to intolerance, two due to clinical deterioration and one due to planning to become pregnant. Longer‐term studies are needed, particularly in light of the potential for dependence. Funding: No funding reported. Cannabis supplied by Tikun Olam, Israel.
van de Donk 2019	To explore the analgesic effects of inhaled pharmaceutical‐ grade cannabis in patients with chronic fibromyalgia pain.	RCT, controlled 4‐ way crossover with measures of experimental pain	Netherlands *n* = 20 F; Average age 39 ± 3 years Patients with chronic fibromyalgia pain.	22% THC (220 mg/g), < 1% CBD Dried, milled, homogenised flower; Inhalation, vaporised; 100 mg (22.4 mg THC, < 1 mg CBD) During 4 experimental sessions, each participant received one of the four interventions with a 2 week washout period.	6.3% THC, 8% CBD (63 mg/g THC, 80 mg/g CBD); 200 mg (13.4 mg THC, 17.8 mg CBD) 9% CBD (90 mg/g CBD), < 1% THC; 200 mg (18.4 mg CBD, < 1 mg THC) Placebo	Nil effect on experimental pain measures of spontaneous or electrical pain responses. Significant increased pressure pain threshold (another experimental pain measure) with THC containing products compared to placebo. Adverse events reported with the THC 22% product included drug high (*n* = 16; 80.0%), coughing (*n* = 14; 70.0%), bad taste (*n* = 5; 25.0%), dizziness (*n* = 3; 15.0%), nausea (*n* = 3; 15.0%), sore throat (*n* = 2; 10.0%), headache (*n* = 1; 5.0%), ‘sleepy’ (*n* = 1; 5.0%). No SAE. Similar adverse effects were described for the other cannabinoid containing products. Funding: Bedrocan international BV supplied the cannabis and Volcano device.
Weizman 2018	To characterise functional brain changes with delta‐9‐THC in patients with chronic neuropathic pain.	RCT, crossover	Israel *n* = 15 M; Mean age 33.3 ± 3.9 years Chronic lumbar radicular pain	Based on the clinical trial registration NCT02560545 Sublingual cannabis oil (20% THC) 0.2 mg/kg, average THC dosage = 15.4 ± 2.2 mg Single dose of cannabis product or placebo in two experimental sessions in a counterbalanced within subjects crossover design with a washout period of at least a week	Placebo	This is a graph theory‐based study using functional magnetic resonance imaging. A significant reduction in pain was reported, along with a correlation with reduced functional connectivity between the cognitive‐affective modulation areas of the brain and the sensorimotor cortex. This study provides insights into the neural mechanisms involved in analgesia, suggesting cognitive‐affective integration interference. Funding: Yahel Foundation, Recanati, New York and Ministry of Science, Technology and Space.

Abbreviations: CBD, cannabidiol; CBN, cannabinol; COPD, chronic obstructive pulmonary disease; MDI, metered dose inhaler; RCT, randomised controlled trial; THC, delta‐9‐tetrahydrocannabinol.

* The composition of products reported in published studies is replicated in this Table.

^^^ Percentages are reported as whole numbers in the published paper.

**TABLE A2:** Observational trials—Safety (*n* = 9).

Citation	Aims or objectives	Study design	Location and sample characteristics	Cannabis product consistent with Category 5*	Comparator (or comparison products)	Main safety findings (focused on cannabis products consistent with Category 5)
Aviram 2022	To retrospectively analyse real world data to assess the potential effectiveness and safety of medical cannabis administered via MDI.	Observational (retrospective analysis of data collected in a free patient support program)	Israel *n* = 143; M 54%; Mean age 62 ± 17 years Chronic neuropathic pain 72.0%, chronic musculoskeletal pain 9.8%, cancer pain 6.3%, chronic nociplastic pain 2.8%, chronic visceral pain 1.4%, medical conditions with concomitant chronic pain 4.2%, conditions other than chronic pain 3.5%	22% THC** Granulated dried flower; Inhalation via MDI; 0.25 mg and 0.5 mg THC 1–4 doses daily initially (titration plan based on previous use, age and comorbidities) + when required doses Adverse events were followed up for 15 months.	Nil	Study withdrawals due to adverse events (*n* = 3) or ineffectiveness (*n* = 5) occurred prior to ‘achieving a balanced regimen’. Most adverse events were reported during the titration phase (33.5%) decreasing to ≤ 4% at 3–15 months. Adverse events included nervous system disorders 23.1% (dizziness, confusion, sleepiness, concentration impairment, memory impairment, headache), gastrointestinal disorders 9.7% (nausea, heartburn, dry mouth, vomiting), psychiatric disorders 10.5% (anxiety, restlessness), cardiovascular disorders 1.4% (palpitations), miscellaneous disorders 12.6% (cough, tinnitus, muscle pain). During follow‐up, 28 patients withdrew from the patient support program, including five due to ineffectiveness and one due to adverse events. Out of the 59 patients reporting adverse events with the inhaler, 50.8% retrospectively reported adverse events from previous medical cannabis use. Non‐MDI medicinal cannabis was continued by *n* = 12 patients during the study. Funding: Syqe Medical Ltd.
Bar‐Sela 2019	To evaluate the dosage‐ controlled cannabis capsules for cancer‐related cachexia and anorexia syndrome in patients with advanced cancer.	Pilot study (clinical setting)	Israel *n* = 24 (7 withdrew before cannabis intake, 11 participated for more than 2 weeks and 6 completed the study); M 62.5%; Median age 66 years Cancer‐related cachexia and anorexia syndrome in advanced cancer	NCT02359123 *n* = 4 patients started on a 10 mg dose, *n* = 13 were started on a dose of 5 mg (4.75 mg THC, 0.25 mg CBD) oral capsules Up to 2 capsules daily; up to 6 months	Nil	17.6% of patients (*n* = 3/17) had a ≥ 10% weight increase with one or two 5 mg capsules daily. At study completion, 83.3% (*n* = 5/6) reported increased appetite. Three out of four patients receiving a 10 mg dose withdrew due to adverse effects (e.g., tiredness, dizziness, disorientation, anxiety, hallucinations, and impaired general functioning). 23.1% of individuals (*n* = 3/13) who received 5 mg capsules withdrew due to similar adverse effects. All psychoactive adverse events occurred 1–2 h post‐administration, for a duration of 2–3 h and caused incapacity to be physically active. Overall, adverse events were mild to moderate. Funding: Cannabics Pharmaceuticals Inc.
Brunt 2014	To assess patient therapeutic satisfaction and subjective effects of prescribed pharmaceutical grade cannabis products with different cannabinoid compositions.	Observational (cross‐sectional survey) – Survey distributed following pharmacy supply of prescribed medicinal cannabis	Netherlands *n* = 102; M 50, F 52; Average age (SD) 52.8, (12.3) years Chronic pain (52.9%), multiple sclerosis (22.5%), cancer (10.8%), ‘psychologic’ problems (7.8%), nausea (5.9%)	‘THC high’ 19% THC, < 1% CBD Inhaled (81.4%) by smoking or via vaporiser or consumed as a tea Mean daily cannabis flower dose (SD) 0.7 g (0.6)	‘THC medium’ (12% THC, < 1% CBD) ‘THC low’ (6% THC, 7.5% CBD)	Higher level of dejection and anxiety with ‘THC high’ compared to ‘THC low’. Funding: The Dutch Ministry of Health, Welfare and Sport.
Crescioli 2020	To describe spontaneously reported adverse events in patients with exposure to medical cannabis.	Observational (Italian Phytovigilance database)	Italy *n* = 53 cases; M 12, F 41; Mean age (SD) 61.9 (15.9) years Total of *n* = 118 adverse events reported between 2006–2018 Neuropathic pain (43.4%) and chronic or non‐ specified pain (39.6%), fibromyalgia (*n* = 5), multiple sclerosis (*n* = 2)	22% THC** Dried flower; Predominantly oral administration (*n* = 1 inhalation) Mean cannabis dose of 138.5 (±SD, 139.0) mg per day; Length of exposure 1 day to 37 months (Mean 149.3 ± 242.4 days).	6.3% THC, 8.8% CBD** Standardised concentrations (5%–8% THC, 7.5%–12% CBD)** Cannabis inflorescences, and magistral preparations.	Fifty‐three cannabis‐related spontaneous adverse event reports were analysed, of these 24 were related to 22% THC (non‐magistral preparations) and this subset is included in ‐ needs to be removed hereafter. Sixteen cases were nonserious, seven were serious and the classification was not recorded for one case. Twenty‐one cases showed improvement or complete resolution. Twenty‐three cases were judged as probably related to cannabis. Drowsiness (*n* = 5), mental confusion (*n* = 4), dizziness (*n* = 3), balance disorder (*n* = 3) and tachycardia (*n* = 3) were the top five adverse events reported in the subset. Funding: No grant funding from agencies in the public, commercial or not‐for‐profit sectors. The Italian Phytovigilance system is coordinated by the National Institute of Health.
Eisenberg 2014	To explore the pharmacokinetics, safety, tolerability, efficacy, and ease of use of a cannabis MDI in patients with chronic neuropathic pain.	Phase 1a, open‐label	Israel *n* = 8; M 5, F 3; Mean age (SD) 42 ± 14 years Patients with chronic neuropathic pain (stable analgesic regimen that included medicinal cannabis)	19.9% THC, 0.1% CBD, 0.2% CBN inhalation via MDI; single 15.1 ± 0.1 mg dose (containing 3.1 mg ±0.02 mg THC)	Nil	Light headedness (*n* = 7) was reported but was tolerable and required no intervention. A borderline reduction in mean systolic blood pressure was also reported. Pharmacokinetic data is not reported here. Funding: Syqe Medical Ltd.
Moreno‐Sanz 2022	To investigate the effectiveness and safety of THC‐ predominant cannabis flowers inhaled via vaporiser in patients with treatment‐ resistant health conditions enrolled in Project Twenty21 (T21).	Observational (multi‐center registry)	United Kingdom *n* = 344; M 267, F 76, non‐binary 1; Mean age (SD) 38.4 ± 10.4 years Patients registered in T21 (between August 2020 and June 2022): Chronic pain 50.9%, anxiety‐related disorders 25.3%, ADHD 7.0%, PTSD 6.1% and insomnia 2.9%	20% w/w THC, < 1% w/w CBD Cannabis flower; Inhalation via vaporiser	Nil	Adverse events included a mild headache (*n* = 1) and a cannabis naïve patient reported transient memory loss. Funding: No external funding. Three authors are employees of a cannabis company or subsidiary clinic that funded Project T21.
O'Brien 2023	To investigate medicinal cannabis safety, tolerability and effectiveness in patients enrolled in T21.	Observational	Australia *n* = 278; M 48.2%, F 50.7%, non‐binary 1.1%; Average age 39.2 years Patients registered in T21 (between February 2022 and 30 August 2022): chronic pain 49.3%, anxiety 32.4%, PTSD 11.3%, multiple sclerosis 2.8% and other 4.2%.	High THC flower products (20% w/w to 22% w/w THC), < 1% w/w CBD Inhalation, vaporised 0.5 g up to 0.9 g daily	High CBD oils or tablets Balanced CBD:THC oils High THC oils or tablets	Following high THC flower (THC 22% w/w, CBD < 1% w/w) use for a week, one patient experienced increased anxiety 10–15 min post use for a few hours. Medicinal cannabis use was discontinued. Three weeks after commencing a high THC flower product (THC 20% w/w, CBD < 1% w/w) another patient experienced increased anxiety with some paranoid features and muscle heaviness for 2 h. The patient's medicinal cannabis regimen was subsequently changed. A third patient experienced an adverse event that is not reported here with a high THC oil and high CBD oil. No serious adverse reactions were reported. Funding: T21 is supported by medicinal cannabis companies (e.g., Adelaide Compounding Pharmacy Pty Ltd., Beacon Medical Australia Pty Ltd., Cann Global Pty Ltd., Levin Health Ltd), conducted by a Releaf Group Ltd. subsidiary and participants were recruited from Releaf clinics. No author funding was reported for the research and publication. Two authors declared employment and shares with Releaf Group Ltd.
Stack 2023	To evaluate quality‐of‐life outcomes, effectiveness and adverse events with various medicinal cannabis formulations.	Observational (Medicinal cannabis clinics)	Australia *n* = 568 (*n* = 19 THC only); M 264, F 304; Median age (IQR) 48 (24) years Total of *n* = 1314 adverse events reported Participants enrolled in Cannabis Access Clinics Observational Study, using medicinal cannabis for an anxiety disorder (between September 2018 and June 2021): Anxiety type PTSD 27.8%, unspecified 72.2%	THC‐only: THC dose mg/day, median (IQR) 33.0 (19.3) Oral liquid or capsule formulations	CBD‐only CBD‐ or THC‐dominant formulations Balanced formulations	Adverse events included gastrointestinal disorders 23.9% (dry mouth 21.1%, nausea 2.8% (authors note dry mouth and nausea are unlikely to be clinically significant)), psychiatric 39.4% (somnolence 18.3%, anxiety 4.2% (authors note no significant association between anxiety and THC dose), confusion 1.4%, disorientation 1.4%, depression 4.2%, paranoia 1.4%, euphoria 4.2% (highest proportion in the THC‐only group), hallucination 1.4%, psychosis 1.4%, mood disorders and disturbances 1.4%), nervous system disorders 4.2% (dizziness 4.2%), metabolism disorders 8.5% (increased appetite 8.5%), general disorders and administration site conditions 14.1% (fatigue 5.6%, balance problems 2.8%, spaced out feeling 5.6%), eye disorders 5.6% (dry eyes 5.6%), other (undefined) 4.2%. If a participant changed to a different formulation during the study, their adverse event outcomes were included in both formulation categories. Funding: Applied Cannabis Research (A division of Southern Cannabis Holdings).
Vulfsons 2020	To evaluate the usability, feasibility, satisfaction and safety of a medical cannabis MDI, in hospitalised patients. Evaluation of efficacy was not a primary study aim.	Pilot open‐label, proof‐of‐concept	Israel *n* = 21; M 10; Mean age ± SD (range), 44.3 ± 12.5 (25–78) years Hospitalised patients with a valid licence for medical cannabis (between January 2016 and July 2017): Cause of chronic neuropathic pain – oncologic pain and nausea 42.8%, neuropathic pain 42.8%, Crohn's disease 4.8%, multiple sclerosis with spasticity 9.5%	22% THC, 0.1% CBD, 0.2% CBN Granulated dried flower; Inhalation via MDI 4 daily doses 0.5 mg THC plus an additional four ‘SOS’ doses; Median amount THC inhaled per day of 1.5 mg.	Nil	Mild post‐inhalation cough was reported by three participants which resolved spontaneously and there were no severe adverse events. Funding: Syqe Medical Ltd.

Abbreviations: ADHD, attention deficit hyperactivity disorder; CBD, cannabidiol; CBN, cannabinol; IQR, interquartile range; MDI, metered dose inhaler; PTSD, post‐traumatic stress disorder; THC, delta‐9‐tetrahydrocannabinol.

* The composition of products reported in published studies is replicated in this Table.

** Cannabis product name, but not cannabinoid composition reported in study. Additional resources have been consulted to verify cannabinoid composition [21, 54].
