# Supplementary material for: Higher THC Concentration Medicinal Cannabis Products Efficacy and Safety Considerations: A Rapid Review
Source: Drug Alcohol Rev. 2026 Apr 1;45(4):e70145. doi: 10.1111/dar.70145 (PMC13040133; doi:10.1111/dar.70145)
Supplement: Supplementary file 1 — Table S1: Inclusion and exclusion criteria. [file DAR-45-0-s002.docx]

**Table S1. Inclusion and exclusion criteria**

| **Criteria** | **Inclusion** | **Exclusion** |
| --- | --- | --- |
| Population | - Human studies - Adults (> 18 years old) | - Animal studies - Healthy volunteers^**^ |
| Study focus | Examining the efficacy and/or safety of THC products for physical and/or mental health conditions | - Pharmacokinetic parameters^**^ - Extraction or detection methods^**^ - Drug seizures - Driving^**^ |
| Interventions | Products consistent with the composition of Category 5 medicinal cannabis products^*^ as defined by the TGA (e.g. proportion of cannabidiol content to total cannabinoid content) including:   - Extract 75% to 88% THC - Dried flower 13% to 60% THC - Inhalation 800 mg/mL to 880 mg/mL THC - Inhalation pressurised 2.5mg to 5 mg THC/actuation - Oral liquid 18 mg/mL to 100 mg/mL THC | Cannabis products with cannabinoid compositions that are not consistent with the Category 5 definition or containing other active ingredients:   - Products where the cannabinoid composition is not clearly specified. - Products consistent with other TGA categories e.g. Category 1 (CBD only), Category 2 (CBD dominant), Category 3 (Balanced THC:CBD ~1:1 products), Category 4 (THC dominant).^**^ - Studies where dose forms are modified so that the final product would not be consistent with a Category 5 product.^**^ - Registered medicinal cannabis products e.g. Sativex (nabiximols), Epidyolex/Epidiolex.^**^ - Synthetic THC and analogues (e.g. dronabinol and nabilone). - Synthetic cannabinoids (e.g. K2, Spice). - Newer hemp derivatives e.g. Delta-8, Delta-10, HHC (unless the study also focuses on high concentration delta-9-THC). - Endogenous cannabinoids (e.g. AEA and 2- AG). - Other phytocannabinoids (e.g. CBG, etc.).^**^ - Sole focus on phytoconstituents e.g. D- limonene, flavonoids. - Sole focus on THC Metabolites (e.g. 11-OH-THC, 11- COOH-THC) or acids (e.g. THCA). |
| Outcomes | - Efficacy (e.g. symptom improvement) - Safety (e.g. adverse events) | Studies that included multiple products with a range of cannabinoid compositions, including but not limited to Category 5 products, that presented aggregate findings.^**^ |
| Study design | RCTs and observational studies | Editorials^**^, commentaries^**^, opinion pieces^**^, book chapters^**^, Letters to the Editor, interviews, case studies^**^, study protocols, in vitro studies, conference abstracts, narrative and systematic reviews^**^ |
| Language | English language publications |  |
| Time Frame | Studies published from 2014 to 30 July 2024 |  |

^*^TGA medicinal cannabis categorisation is based on proportion of cannabidiol to total cannabinoid content. Examples of THC concentration in Category 5 products may vary based on the 6-month reporting period. Consistent with the potency of Category 5 products based on industry reporting between January 2024 and June 2024 (not published at the time of original protocol development) herb, dried 14%-60% was updated to include dried flower 13%-60% and inhalation pressurised 250-500 mg to 2.5-5 mg THC/actuation, and oral liquid from 20 mg/mL-50 mg/mL to 18 mg/mL-100 mg/mL.

^**^Additional exclusion criteria.
