# Supplementary material for: Higher THC Concentration Medicinal Cannabis Products Efficacy and Safety Considerations: A Rapid Review
Source: Drug Alcohol Rev. 2026 Apr 1;45(4):e70145. doi: 10.1111/dar.70145 (PMC13040133; doi:10.1111/dar.70145)
Supplement: Supplementary file 2 — Table S2: Newcastle–Ottawa Scale quality assessment of included observational studies. Table S3: Modified Jadad scores of the included randomised clinical trial studies. [file DAR-45-0-s001.docx]

**Table S2. Newcastle–Ottawa Scale quality assessment of included observational studies**

| **Newcastle–Ottawa Scale quality assessment of included observational studies** | | | | |
| --- | --- | --- | --- | --- |
| **Citation** | **Selection ****** | **Comparability **** | **Outcome ***** | **Total score** |
| Aviram 2022 | ** |  | ** | 4 (low) |
| Bar-Sela 2019 | *** |  | ** | 5 (moderate) |
| Brunt 2014 | *** |  | * | 4 (low) |
| Crescioli 2020 | *** |  | * | 4 (low) |
| Eisenberg 2014 | ** |  | ** | 4 (low) |
| Moreno-Sanz 2022 | *** |  | *** | 6 (moderate) |
| O’Brien 2023 | *** |  | *** | 6 (moderate) |
| Stack 2023 | *** |  | * | 4 (low) |
| Vulfsons 2020 | *** |  | ** | 5 (moderate) |

The Newcastle–Ottawa Scale was used to assess the quality of observational cohort studies. The scale evaluates three domains: selection, comparability, and outcome. The selection domain assesses the adequacy of selecting exposed and non-exposed cohorts, with a maximum of four stars. The comparability domain evaluates whether studies controlled for important comparator and confounding factors, with a maximum of two stars. The outcome domain examines how outcomes were assessed and followed up, with a maximum of three stars. Overall study quality was classified as high (7–9 stars), moderate (5–6 stars), or low (≤4 stars).

**Table S3. Modified Jadad scores of the included randomised clinical trial studies.**

| **Modified Jadad scores of the included randomised clinical trial studies.** | | | | | | | | |
| --- | --- | --- | --- | --- | --- | --- | --- | --- |
| **Citation** | **Randomised (Yes=1, No=0)** | **Randomisation Appropriate (Yes=1, No=-1, Not described=0)** | **Double-Blind (Yes=1, No=0)** | **Blinding Appropriate (Yes=1, No=-1, Not described=0)** | **Withdrawals/ Dropouts Reported (Yes=1, No=0)** | **Allocation concealment (adequate=2, unclear=1, inadequate=0)** | **Total score** | **Quality** |
| Abdallah 2018 | 1 | 1 | 1 | -1 | 1 | 2 | 5 | High |
| Almog 2020 | 1 | 0 | 1 | 0 | 1 | 2 | 5 | High |
| Di Luca 2023 | 1 | 0 | 1 | 1 | 0 | 1 | 4 | Moderate |
| Naftali 2021 | 1 | 0 | 1 | 0 | 1 | 1 | 4 | Moderate |
| Van de Donk 2019 | 1 | 1 | 1 | -1 | 1 | 2 | 5 | High |
| Weizman 2018 | 1 | 1 | 1 | 1 | 1 | 2 | 7 | High |

Scores are based on the Modified Jadad scale, assessing randomisation, appropriateness of randomisation, blinding, appropriateness of blinding, reporting of withdrawals/dropouts and allocation concealment. This scoring adjustment is reflected in total scores ranging from 0–7, with higher scores indicating better methodological quality. Quality categories are: **High (5–7), Moderate (3–4), Low (≤2).**

Scores are based on the Modified Jadad scale, assessing randomisation, appropriateness of randomisation, blinding, appropriateness of blinding, reporting of withdrawals/dropouts and allocation concealment. This scoring adjustment is reflected in total scores ranging from 0–7, with higher scores indicating better methodological quality. Quality categories are: **High (5–7), Moderate (3–4), Low (0–2)**.
